# A novel method using a differential staining fluorescence microscopy (DSFM) to track the location of *enteric* pathogens within mixed-species biofilms

**DOI:** 10.1038/s41598-023-42564-6

**Published:** 2023-09-16

**Authors:** Qiyue Chen, Rong Wang, Joseph M. Bosilevac, Manita Guragain, Sapna Chitlapilly Dass

**Affiliations:** 1 Department of Animal Science, Texas A&M University, College Station, TX 77845 USA; 2 U. S. Department of Agriculture, Roman L. Hruska U.S. Meat Animal Research Center, Clay Center, Lincoln, NE 689330166 USA; 3 U. S. Department of Agriculture, Eastern Regional Research Center, Wyndmoor, PA 19038 USA

**Keywords:** Ecology, Microbial ecology

## Abstract

This study developed a new tool, differential staining fluorescence microscopy (DSFM), to measure the biovolume and track the location of *enteric* pathogens in mixed-species biofilms which can pose a risk to food safety in beef processing facilities. DSFM was employed to examine the impact of pathogenic bacteria, *Escherichia coli* O157:H7 and three different *Salmonella enterica* strains on mixed-species biofilms of beef processing facilities. Fourteen floor drain biofilm samples from three beef processing plants were incubated with overnight *BacLight* stained *enteric* pathogens at 7 °C for 5 days on stainless steel surface then counter-stained with FM-1-43 biofilm stain and analyzed using fluorescence microscopy. Notable variations in biovolume of biofilms were observed across the fourteen samples. The introduction of *E. coli* O157:H7 and *S. enterica* strains resulted in diverse alterations of biofilm biovolume, suggesting distinct impacts on mixed-species biofilms by different *enteric* pathogens which were revealed to be located in the upper layer of the mixed-species biofilms. Pathogen strain growth curve comparisons and verification of *BacLight* Red Stain staining effectiveness were validated. The findings of this study show that the DSFM method is a promising approach to studying the location of *enteric* pathogens within mixed-species biofilms recovered from processing facilities. Understanding how foodborne pathogens interact with biofilms will allow for improved targeted antimicrobial interventions.

## Introduction

Mixed-species biofilms commonly found in food processing facilities are a threat to the safety of food products as they provide an optimal environment for food-borne pathogens to thrive. Among these pathogens, Shiga toxin-producing *Escherichia coli* (STEC) O157:H7 (*E. coli* O157:H7) and *Salmonella enterica* (*S. enterica*) are of particular concern due to their significant public health impact. *E. coli* O157:H7 is a leading cause of hemolytic uremic syndrome, a severe and potentially life-threatening complication^1^, while *S. enterica* is responsible for the most foodborne illness outbreaks in the United States^2^. These pathogens can be transmitted to humans through contaminated food, including meat, poultry, eggs, and fresh produce, highlighting the importance of effective control measures in food processing facilities^3,4^. Of particular concern is the risk to vulnerable individuals, such as young children, elderly people, and those with weakened immune systems^5^. In an effort to prevent food contamination, processing facilities implement a range of control measures. These include stringent sanitation protocols, antimicrobial interventions such as chlorine washing, proper hygiene training for employees, and the application of Hazard Analysis Critical Control Point (HACCP) principles^4,6^. A key strategy among these is temperature control, which involves maintaining low temperatures to inhibit the growth of harmful pathogens throughout the processing, storage, and transportation stages. Biofilms often conceal pathogens responsible for outbreaks, making it difficult to eradicate them from meat processing plants. Previous research has shown that the presence of multiple bacterial species in a biofilm may increase the likelihood of pathogens such as *E. coli* O157:H7 and *S. enterica* continuing to thrive after they have already established themselves in the community^7,8^. There is a potential correlation between the location of the pathogen and their resistance to antimicrobial inactivation in biofilms due to the metabolic activities and physical structure of the biofilms^9^. It has been observed that bacteria in dual-species biofilms are arranged in two distinct layers. *S. enterica* cells are primarily located on the surface of the mixed biofilm, whereas Gram-positive bacteria predominate at the bottom. This strategic positioning of *S. enterica* not only increases competition for scarce resources like oxygen, but also provides a competitive advantage. Moreover, bacterial cells located in the deeper layers of the biofilm are more resistant to antimicrobial treatments due to the biofilm structure's physical barrier.

Thus, the investigation of biofilm structure and pathogen interactions is crucial for ensuring food safety and develop effective hygiene standard in meat processing facilities. Standard light microscopy techniques face challenges in differentiating pathogens within complex multispecies biofilms relevant to food safety^10^. To address the limitations of existing methods, we developed a novel differential staining fluorescence microscopy (DSFM) approach. DSFM enables rapid 3D scanning and improved visualization of pathogens and biovolume. This allows for enhanced accuracy, speed, differentiation, and understanding of pathogen localization compared to other microscopy tools. The simultaneous staining and imaging capabilities provide superior location of pathogen and distribution within mixed-species biofilm structures. DSFM’s applicability to multispecies communities makes it uniquely suited to investigate biofilms from food processing environments. This innovative technique provides critical insights into pathogen persistence within biofilms, which can inform efforts to improve food safety. The present study validated the DSFM method by comparing the growth curves in mixed-species biofilm and verification of dye staining effectiveness. The study findings provide significant insights into the location of *enteric* pathogens within mixed-species biofilms, which will help facilitate the development of pathogen interventions. In this study, three *S. enterica* strains (serovars Cerro, Montevideo, and Typhimurium) and three *E. coli* O157:H7 strains isolated from beef trim, and 14 floor drain biofilm samples collected from three beef processing facilities were used to assess the effectiveness of dyes for staining mixed-species biofilm and *enteric* pathogens.

**Figure 1:**
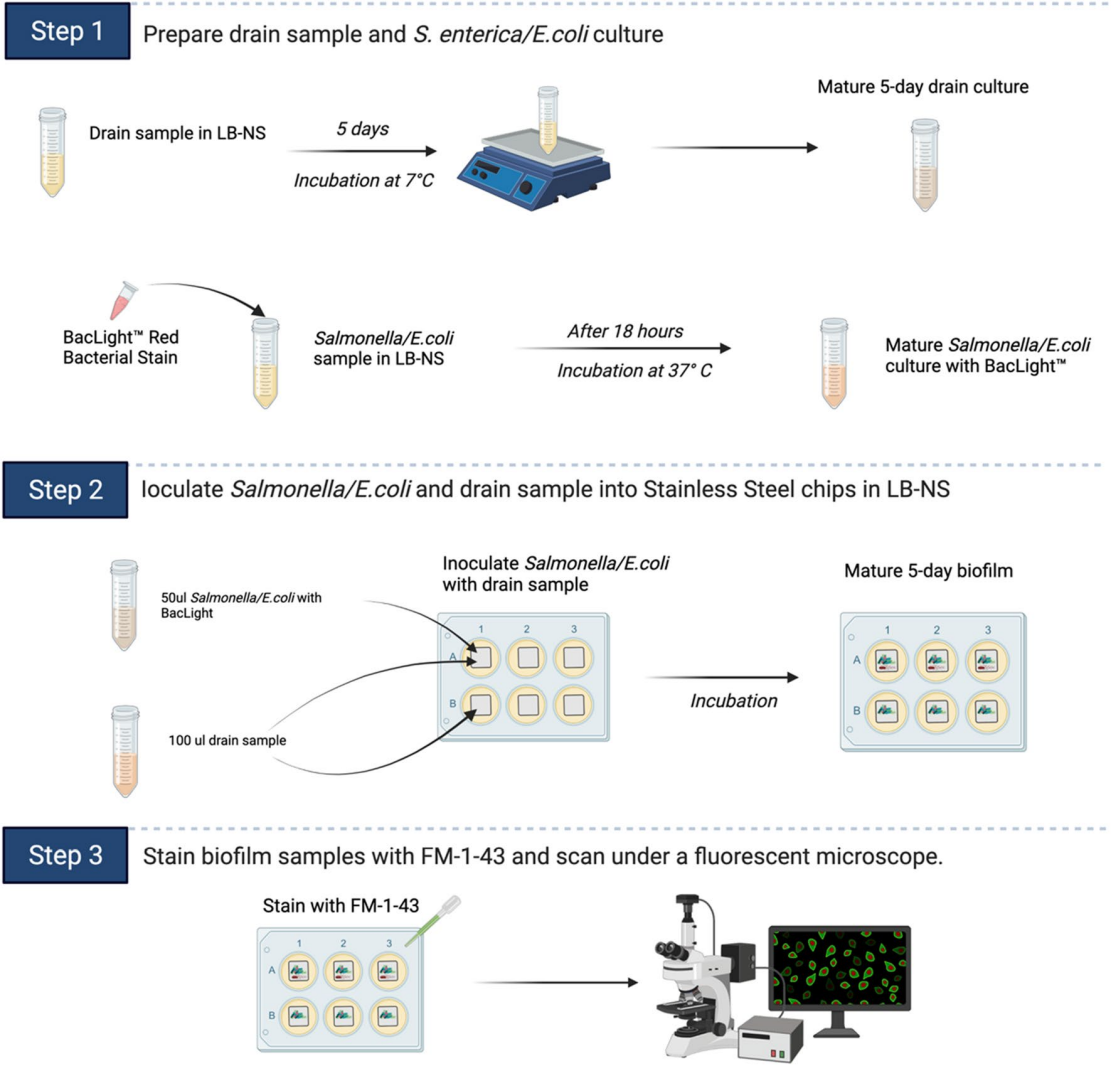
Summary diagram showing the key steps (1–3) in Non-invasive differential staining fluoresce microscopic method to track the location of enteric pathogens within mixed-species biofilm.

For our study, we conducted experiments at a temperature of 7 °C, which is a common temperature in meat processing facilities. By replicating these industry-specific conditions, our study reflects the temperature environments found in these facilities. This is a significant strength of our experiment. By evaluating biofilm formation and pathogen persistence under these realistic conditions, our findings offer valuable insights that are directly applicable to the beef industry. This approach enhances the relevance and applicability of our research, providing valuable insights for the development of effective control measures in the food industry. The results will help dissect the complex mechanisms of multi-species biofilms that form and harbor pathogens, and subsequently lead to improving the elimination of biofilms and reducing the risk of foodborne illnesses (Fig. 1).

## Results

### Spatial organization of mixed-species biofilms observed through fluorescence microscopy

**Figure 2:**
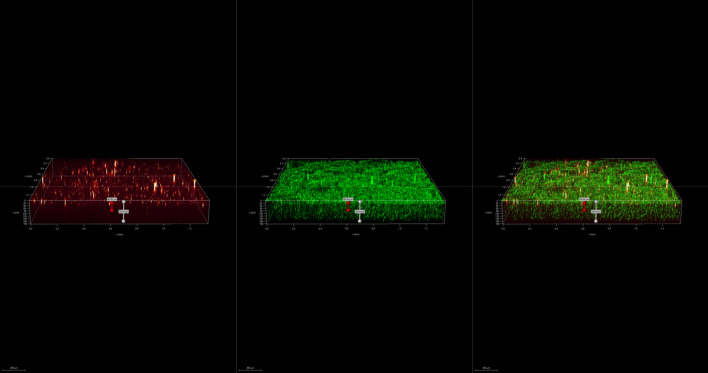
Fluorescence images (magnification 10 ×) of 11A drain biofilm with *E. coli* 110 exposed under: (left) Y5 channel showing *BacLight* stain only; (middle) GFP channel showing FM-1-43 stain only; and (right) overlapping image of both GFP and Y5 channels.

**Figure 3:**
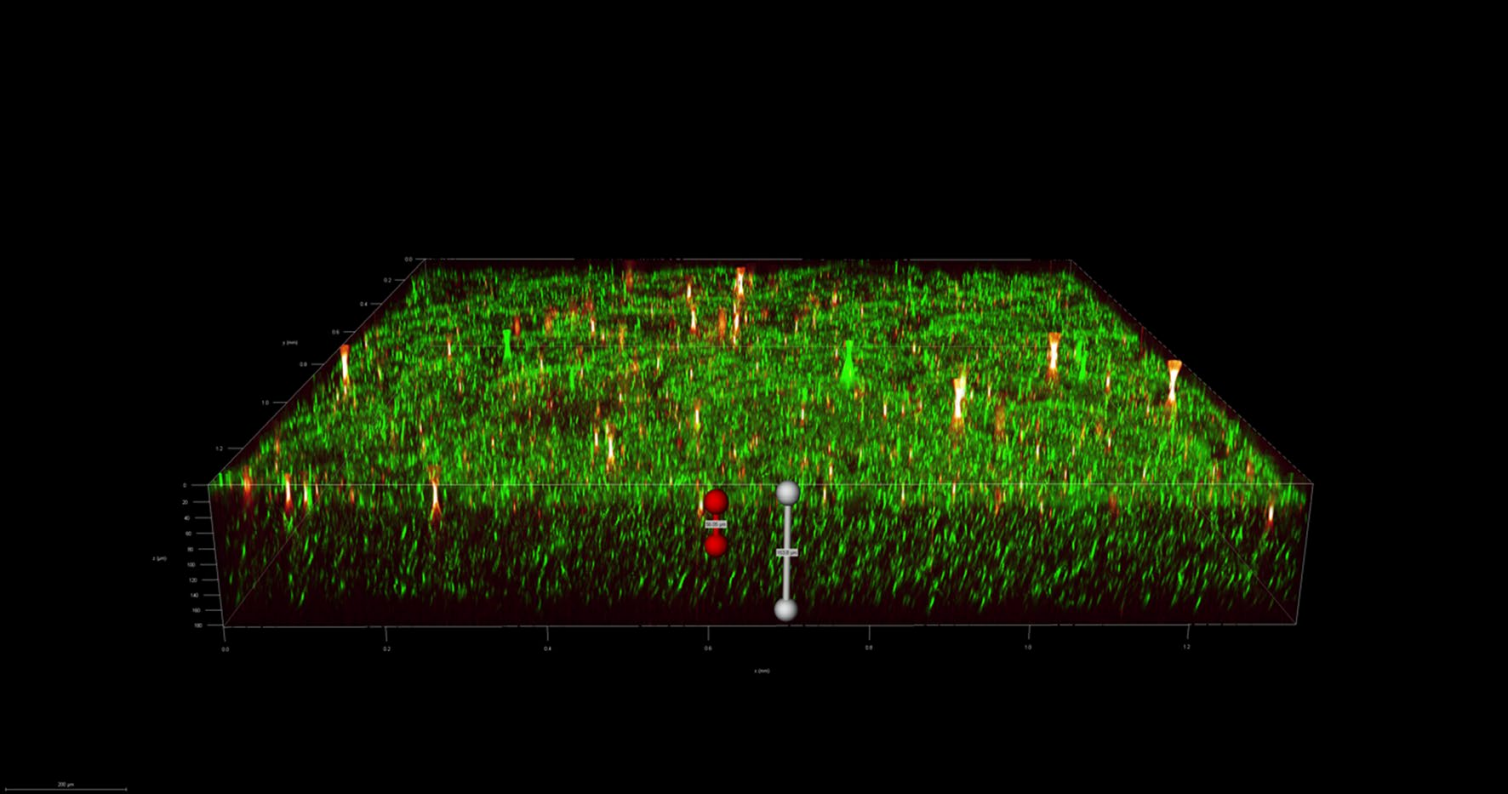
Overlapping fluorescence images (magnification 10 ×) of 11A drain biofilm with three different *E. coli* 110 exposed under both GFP and Y5 channel.

**Figure 4:**
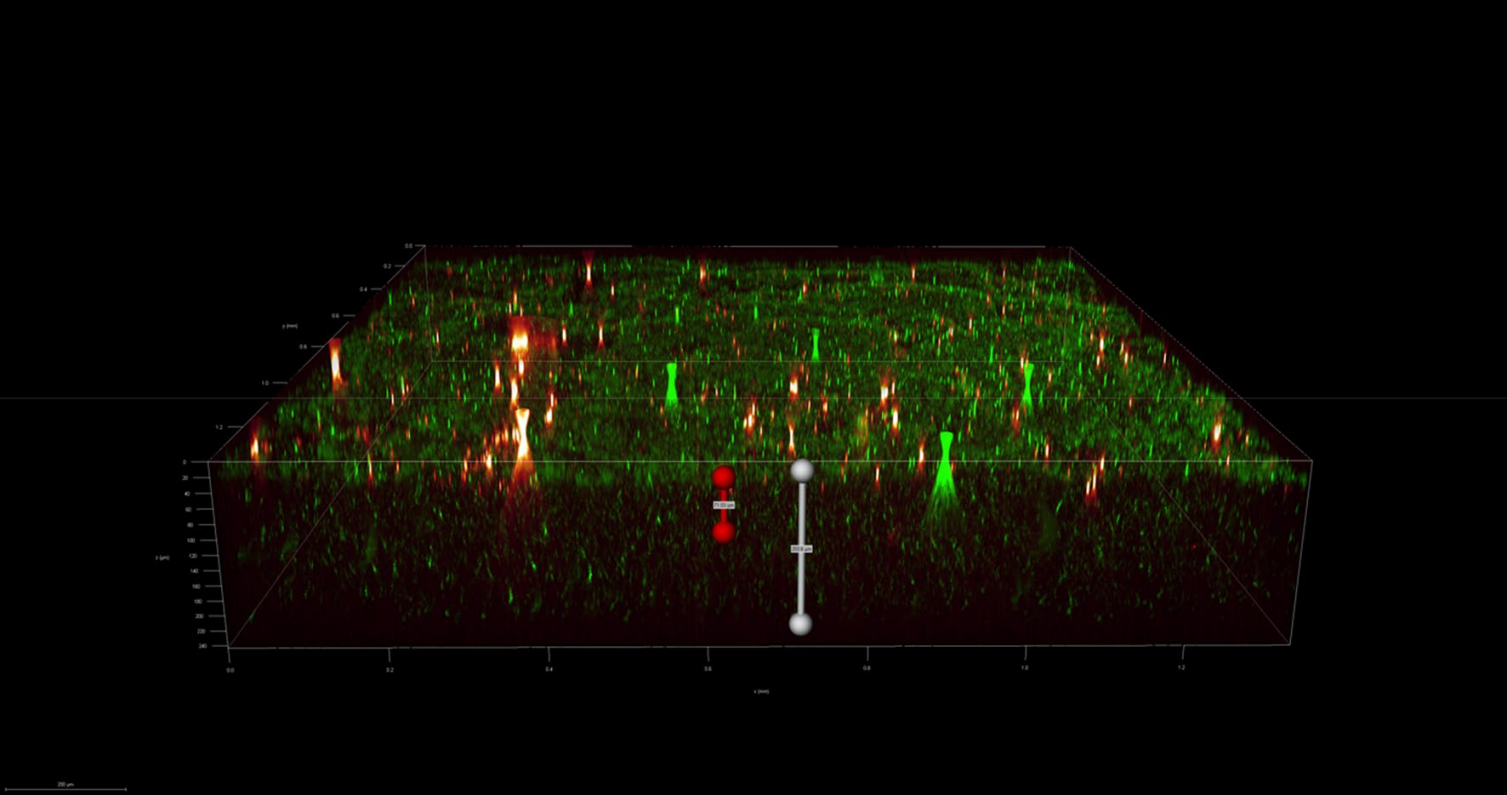
Overlapping fluorescence images (magnification 10 ×) of 11A drain biofilm with three different *E. coli* 138 exposed under both GFP and Y5 channel.

**Figure 5:**
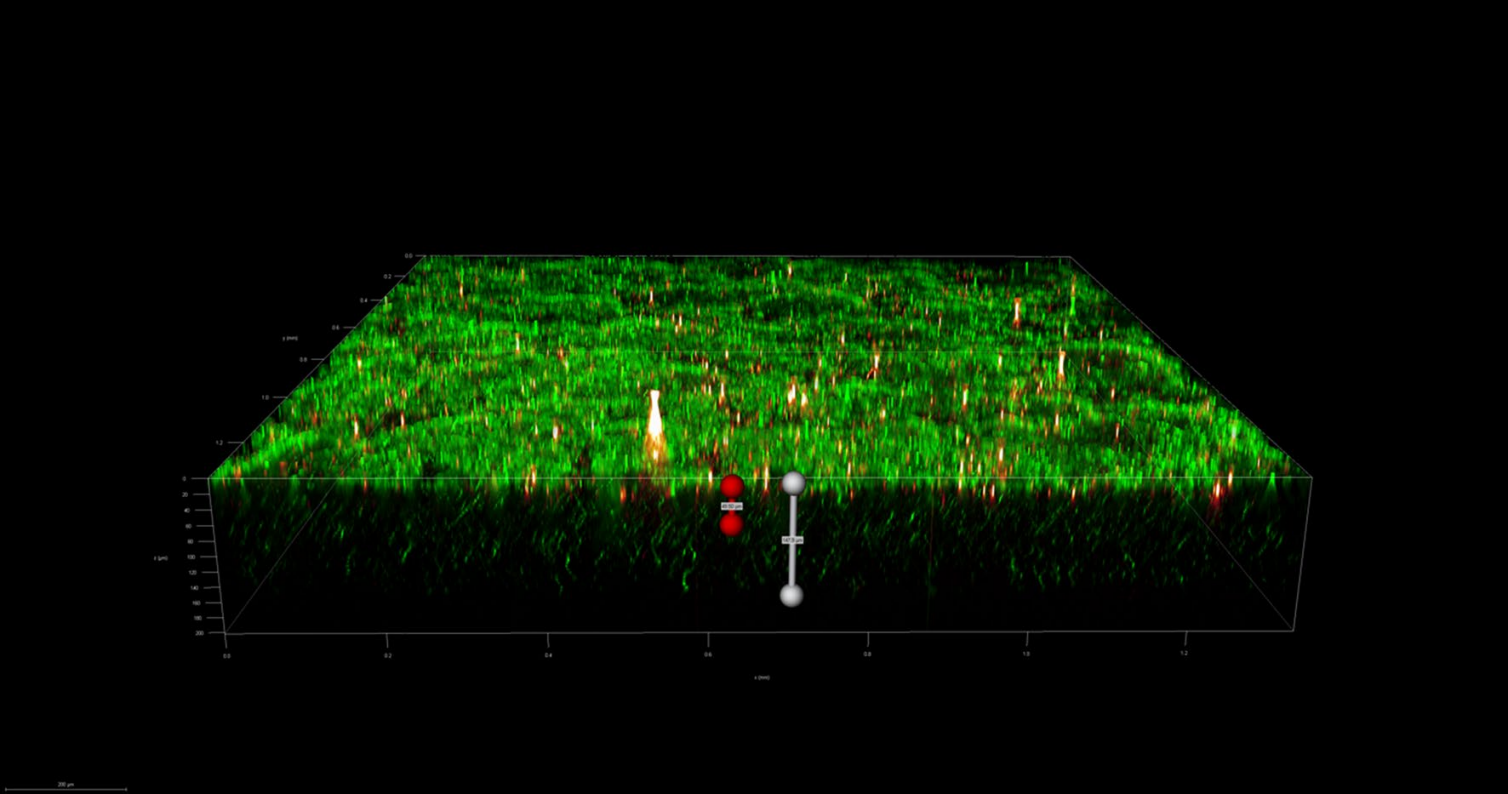
Overlapping fluorescence images (magnification 10 ×) of 11A drain biofilm with three different *E. coli* 141 exposed under both GFP and Y5 channel.

**Figure 6:**
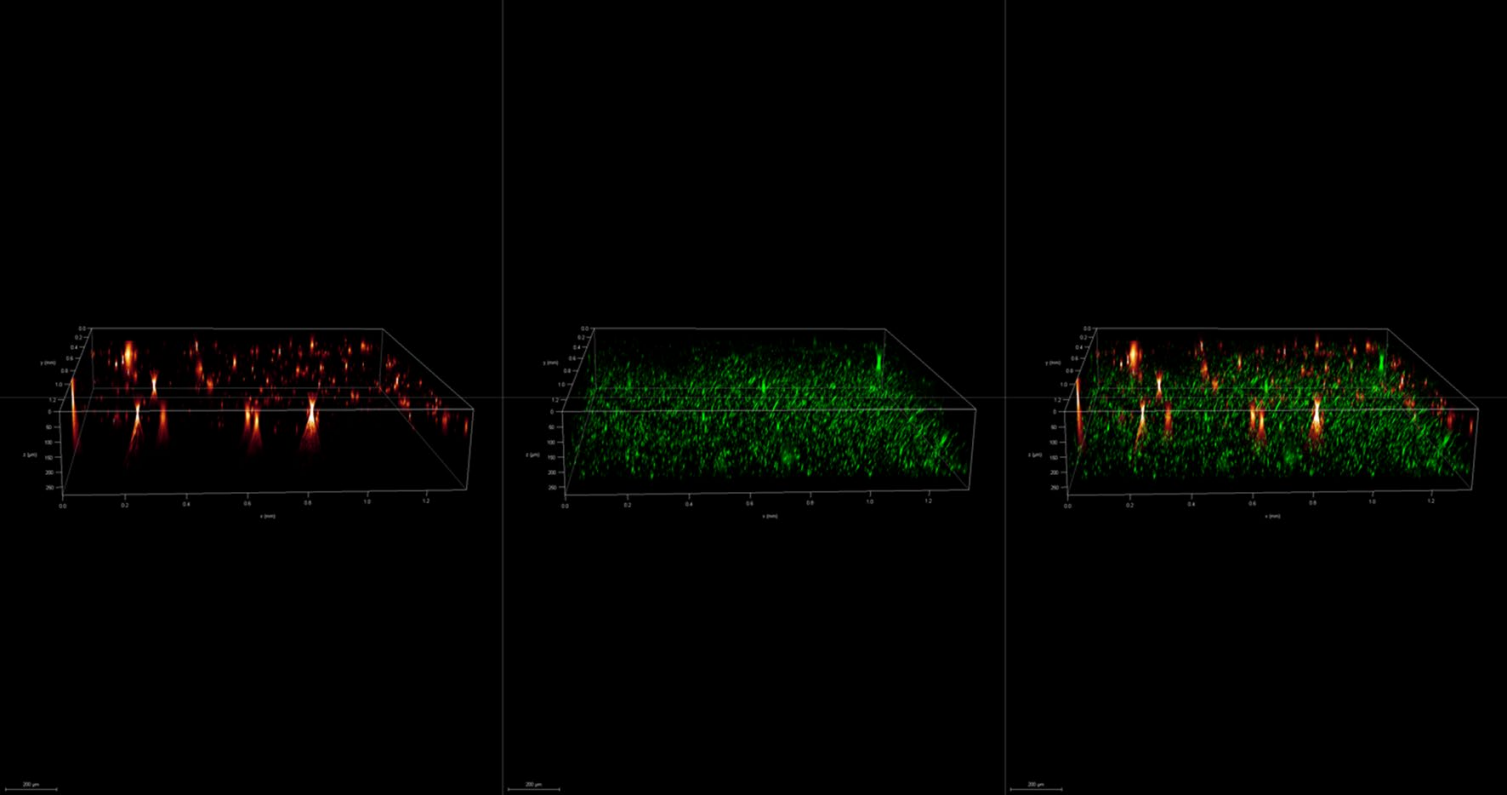
Fluorescence images (magnification 10 ×) of 11B drain biofilm with *E. coli* 138 exposed under: (left) Y5 channel showing *BacLight* stain only; (middle) GFP channel showing FM-1-43 stain only; and (right) overlapping image of both GFP and Y5 channels.

**Figure 7:**
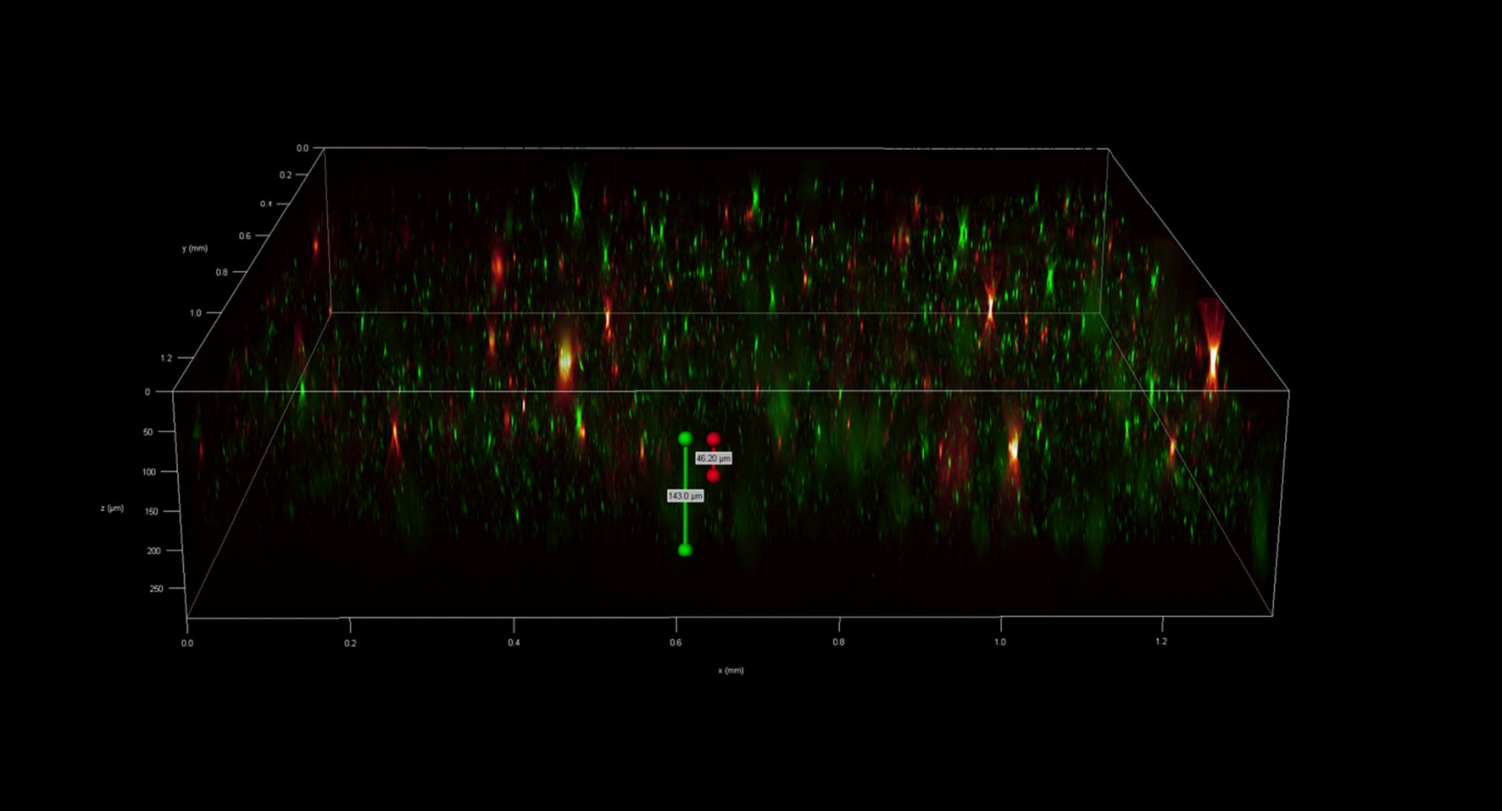
Overlapping fluorescence images (magnification 10 ×) of 11B drain biofilm with three different *E. coli* 110 exposed under both GFP and Y5 channel.

**Figure 8:**
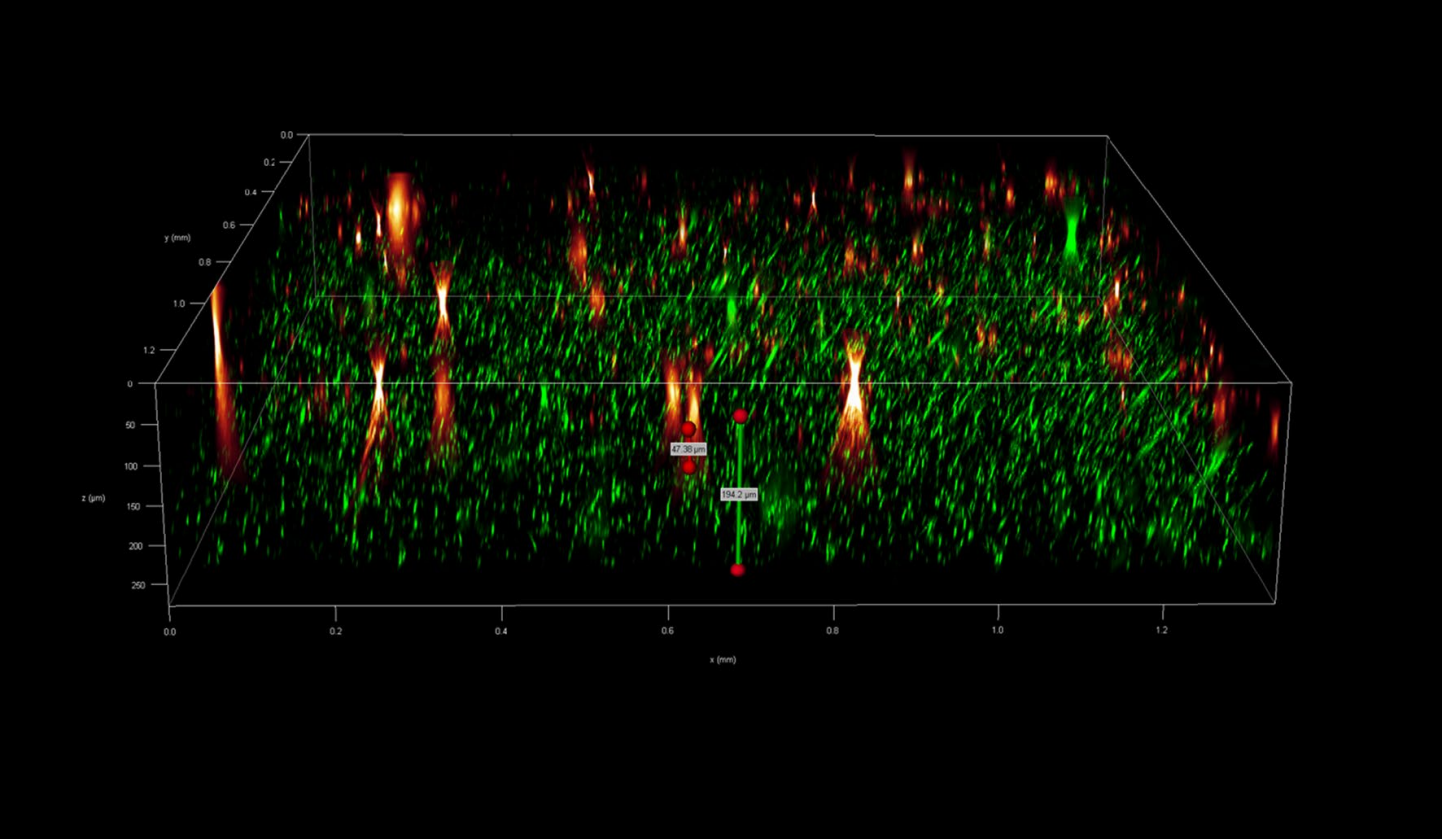
Overlapping fluorescence images (magnification 10 ×) of 11B drain biofilm with three different *E. coli* 138 exposed under both GFP and Y5 channel.

**Figure 9:**
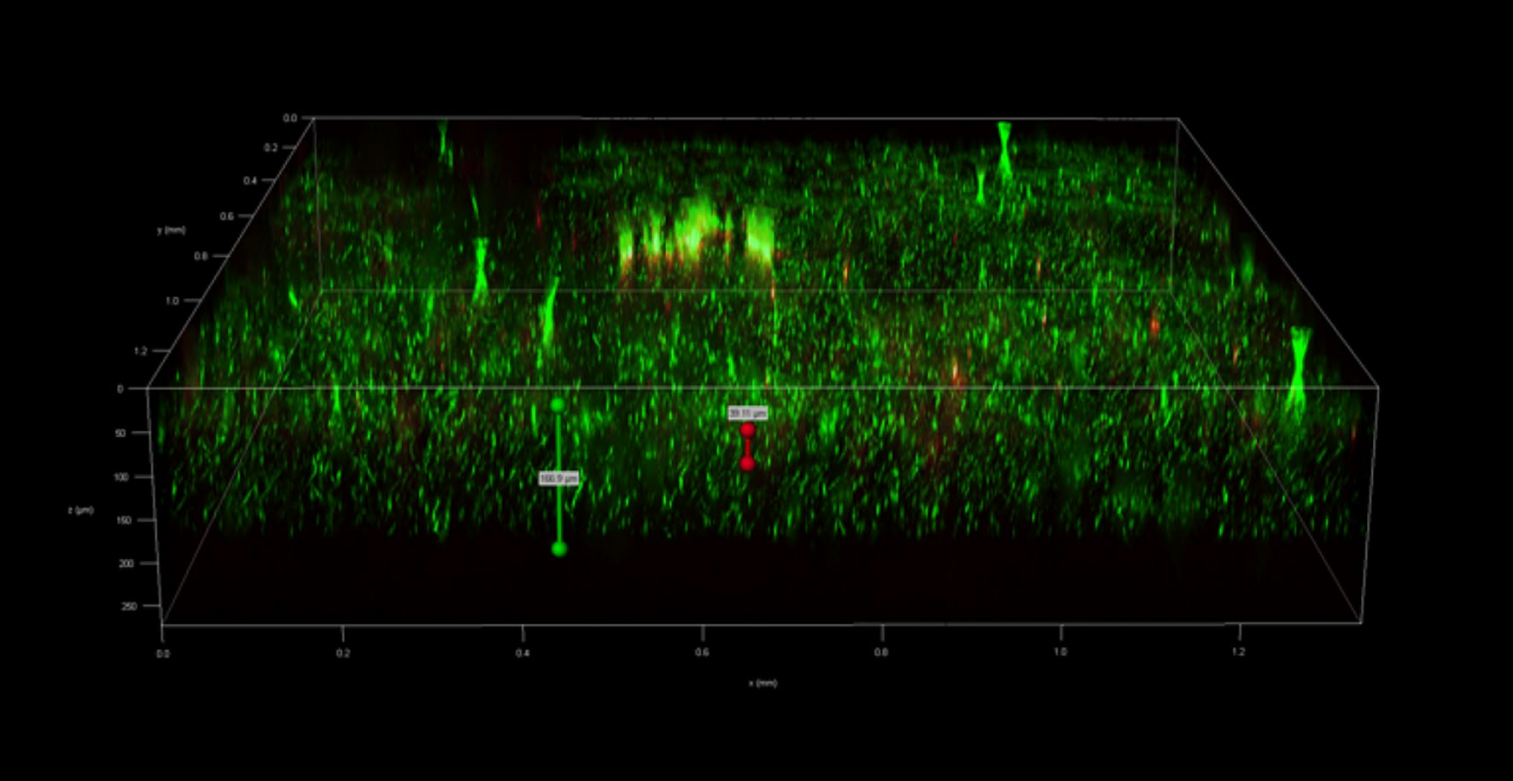
Overlapping fluorescence images (magnification 10 ×) of 11B drain biofilm with three different *E. coli* 141 exposed under both GFP and Y5 channel.

**Figure 10:**
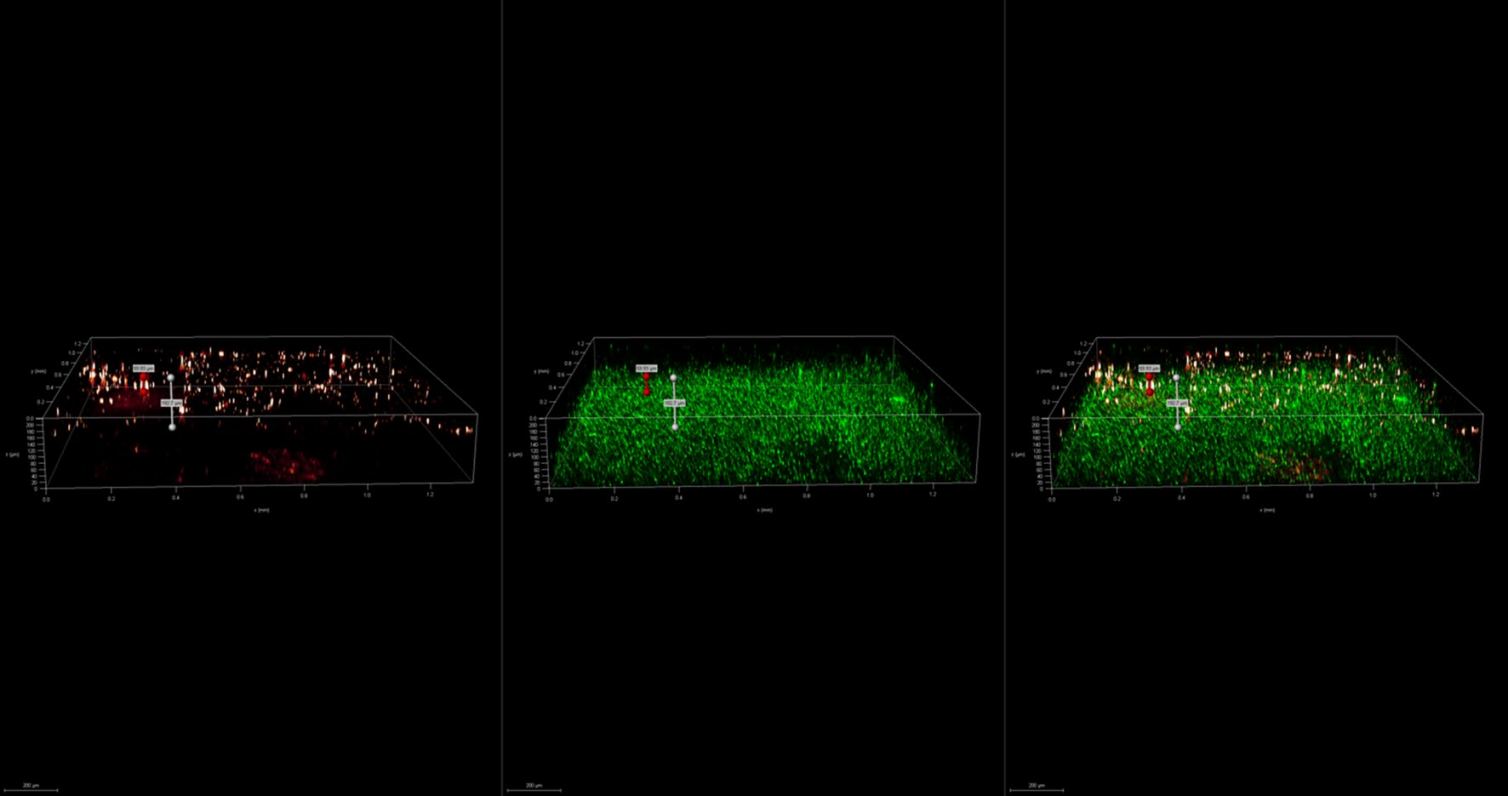
Fluorescence images (magnification 10 ×) of 15C drain biofilm with *S. enterica* serovar Montevideo exposed under: (left) Y5 channel showing *BacLight* stain only; (middle) exposed under GFP channel showing FM-1-43 stain only; and (right) overlapping image of both GFP and Y5 channels.

**Figure 11:**
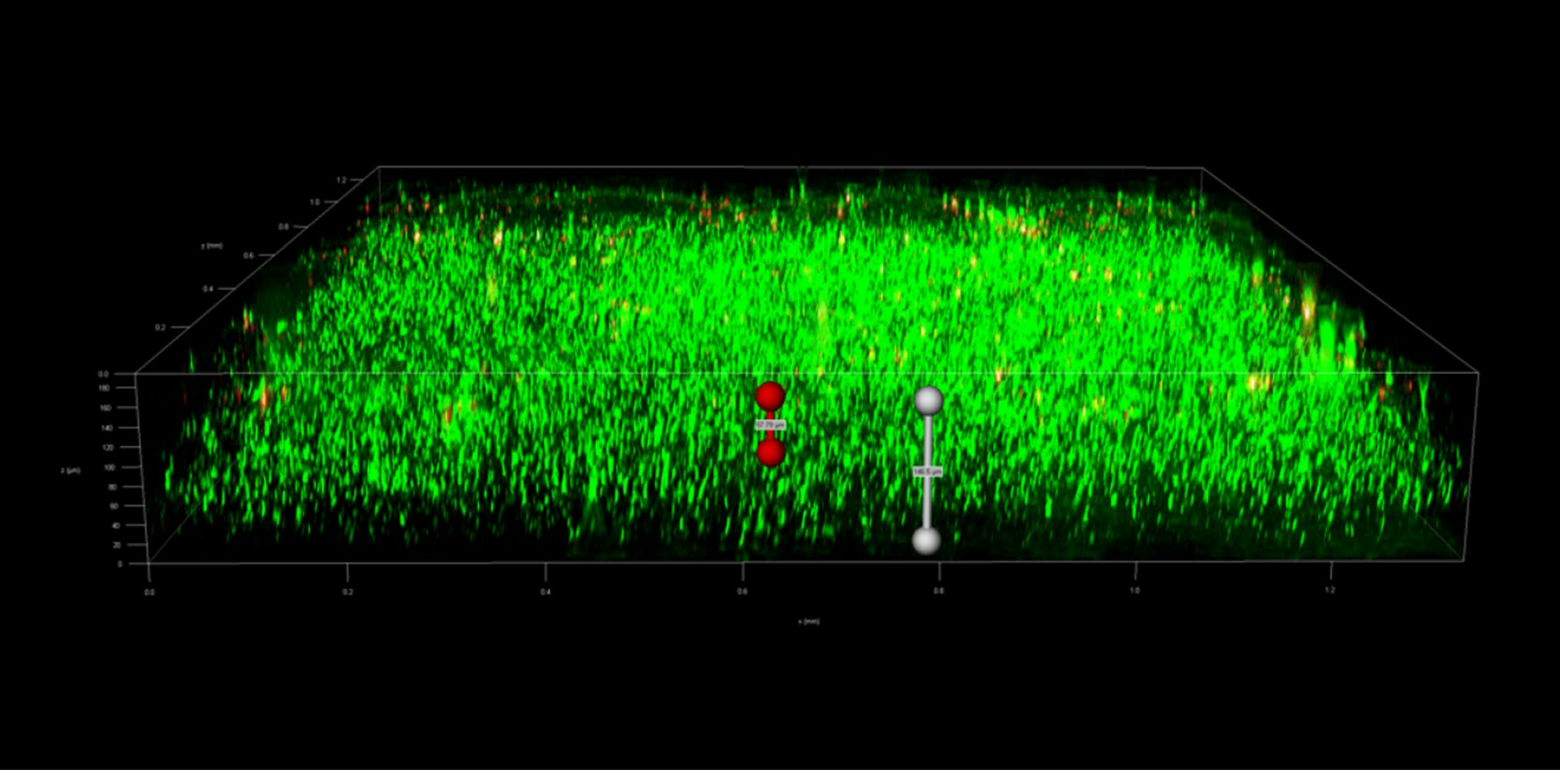
Overlapping fluorescence images (magnification 10 ×) of 15C drain biofilm with three different *S. enterica* serovar Cerro exposed under both GFP and Y5 channel.

**Figure 12:**
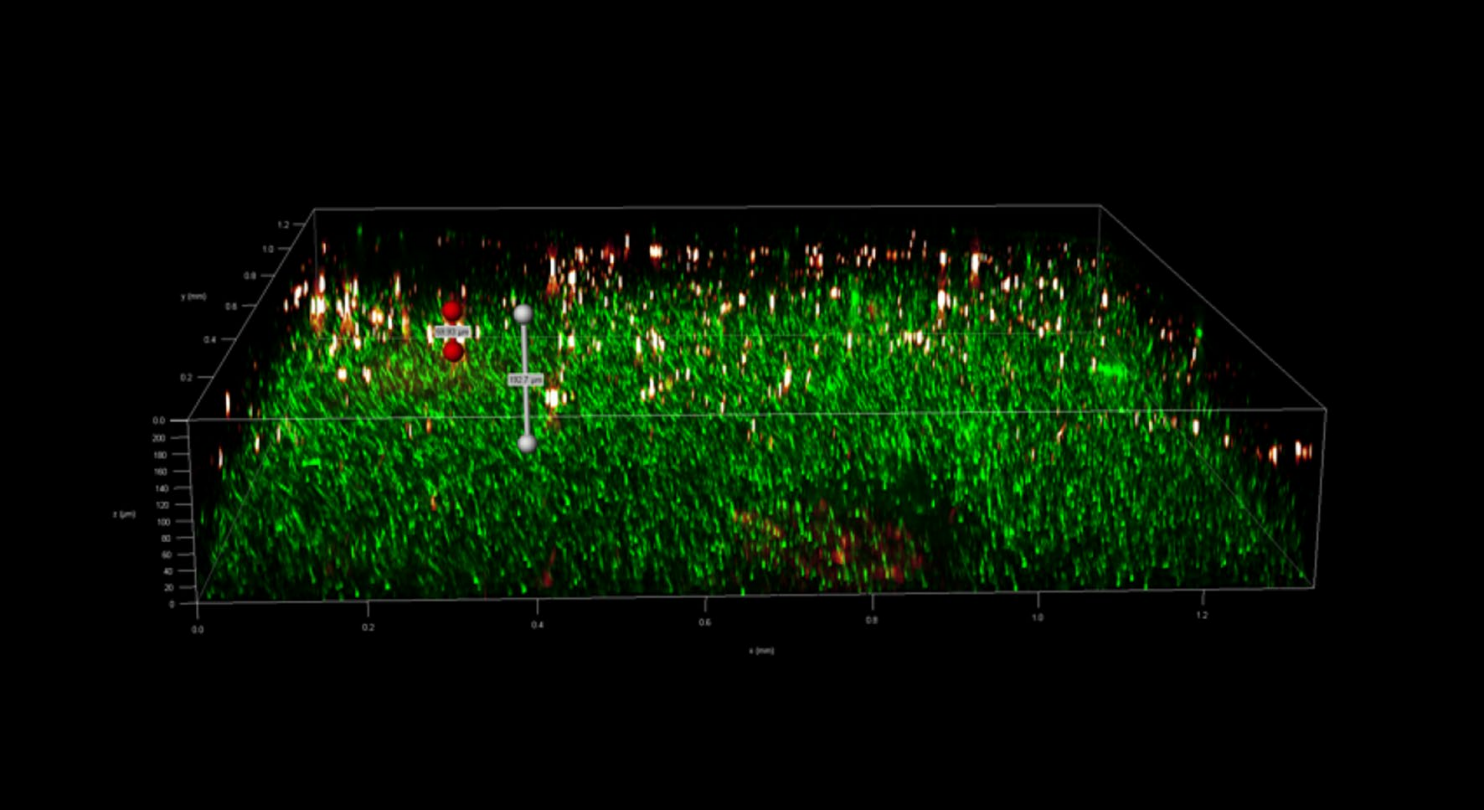
Overlapping fluorescence images (magnification 10 ×) of 15C drain biofilm with three different *S. enterica* serovar Montevideo exposed under both GFP and Y5 channel.

**Figure 13:**
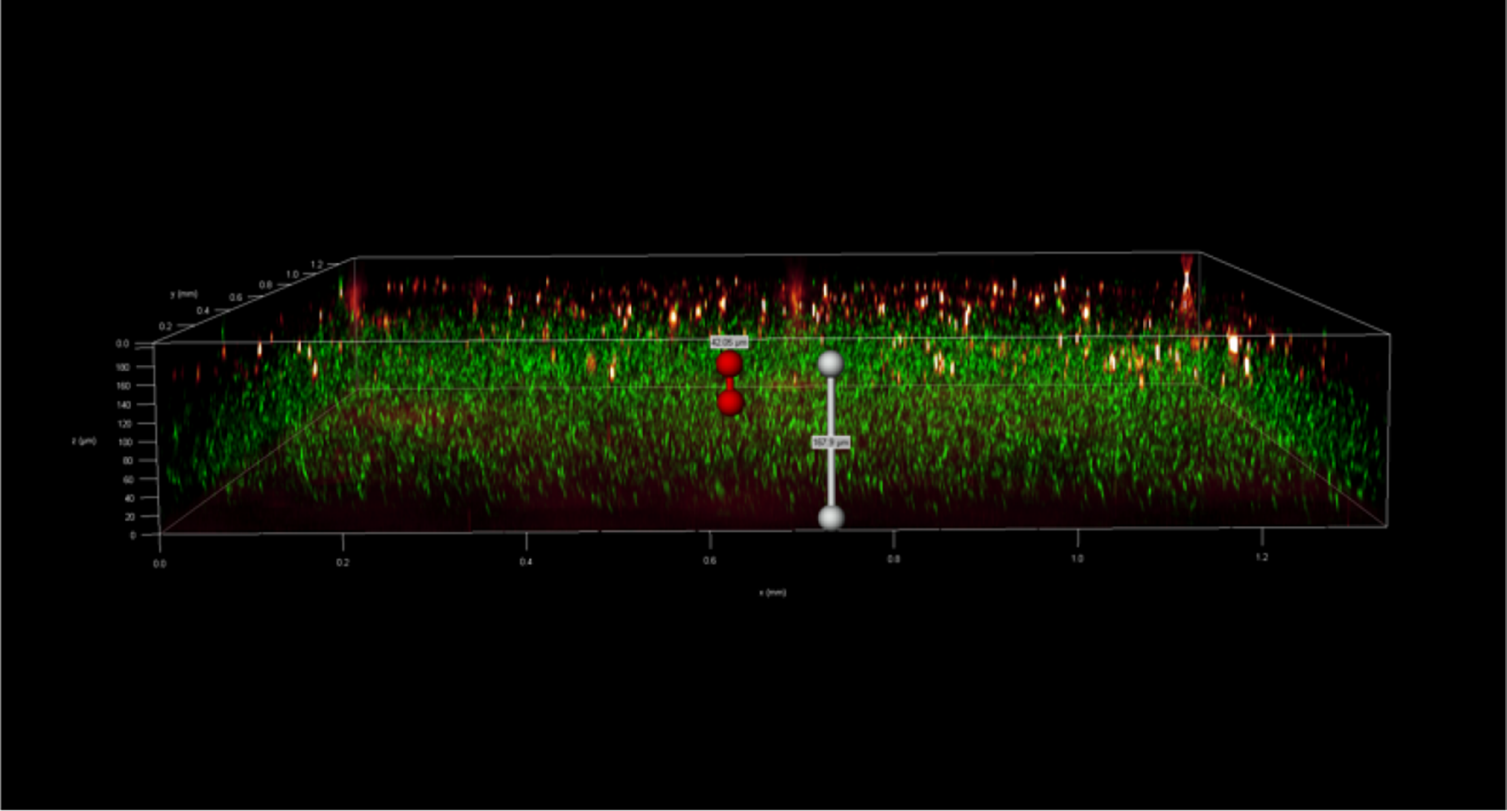
Overlapping fluorescence images (magnification 10 ×) of 15C drain biofilm with three different *S. enterica* serovar Typhimurium exposed under both GFP and Y5 channel.

To investigate the spatial organization of mixed-species biofilms developed on contact surface, drain sample from Plant A, B and C were first inoculated for 5 days. One the final day, *E. coli* O157:H7 and *S. enterica* strain were separately cultured overnight with the *BacLight* Stain, respectively. These pathogen samples were then inoculated with matured 5-day drain samples: *E. coli* O157:H7 with drain samples from Plant A and Plant B drain, and *S. enterica* strain with drain from Plant C. The combinations were incubated for an additional at 7 °C for 5 days on stainless steel (SS) chips. The resulting biofilm was stained with FM-1-43 and subjected to Z-stacking feature in 3D fluorescence microscopy imaging. The results showed that the *S. enterica* and *E. coli* O157:H7 cells were evenly distributed through the upper half of the mixed-species biofilm structure (Figs. 2, 3, 4, 5, 6, 7, 8, 9, 10, 11, 12, and 13).

### Quantification of mixed-species biofilm biovolume for drain sample without S. enterica and E. coli O157:H7 pathogens

**Figure 14:**
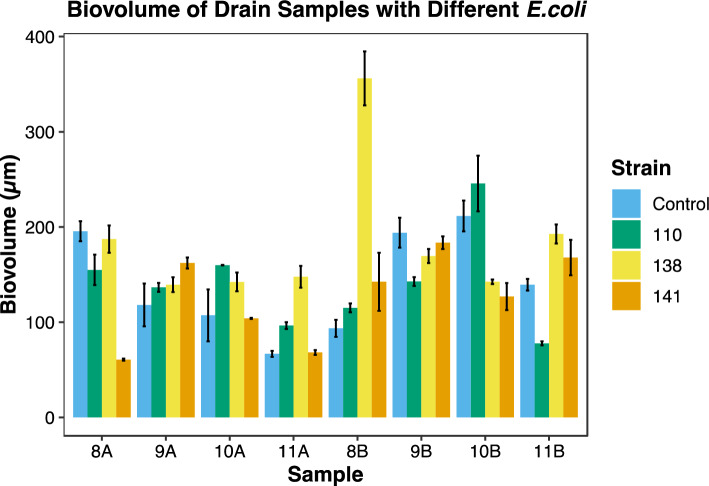
Change of biovolume for drain samples from plant A and B with (*E. coli* O157:H7 110, 138, and 141) and without (Control) enteric pathogen.

**Figure 15:**
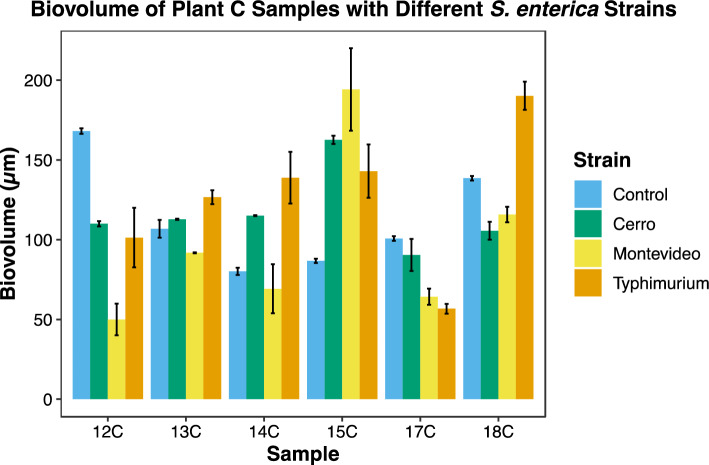
Change of biovolume for drain samples from plant C with (*S. enterica* C-Cerro, C-Typhimurium, and C-Montevideo) and without (Control) enteric pathogen.

Biofilm formation was observed using all 14 drain samples harboring microorganisms at 7 °C on the SS surface. The biovolume of the FM 1:43 stained mixed-species biofilms formed by each drain sample from Plant A and B without *E. coli* O157:H7 varied from 66.8 to 211.6 µm, while the biovolume of FM 1:43 stained mixed-species biofilms formed by each drain sample from Plant C without *S. enterica* ranged from 82.4 to 168.1 µm. Among the samples evaluated, sample 10B had the highest biovolume of 211.6 1, while sample 11A produced the least amount of biovolume at 66.8 µm (Figs. 14 and 15). These findings confirm the ability of microorganisms to form biofilms on SS surfaces under conditions relevant to the food processing industry and highlight the variability in biofilm formation amongst the different samples.

### Quantification of mixed-species biofilm biovolume for drain sample with S. enterica and E. coli O157:H7 pathogens

The biovolumes of the mixed-species biofilms formed on SS surfaces and co-inoculation with *E. coli* O157:H7 or *S. enterica* varied greatly, ranging from 60.8 to 356.1 µm. Specifically, the drain sample 8A co-cultured with *E. coli* 141 had the lowest biovolume of 60.8 µm, whereas the drain sample 8B co-cultured with *E. coli* 138 had the highest biovolume of 356.1 µm. Similarly, the biovolume of the mixed-species biofilms formed by Plant C co-cultured with *S. enterica* had a wide biovolume range between 48.5 and 194.2 µm. In particular, the drain sample 12C with *S. enterica* serovar Montevideo had the lowest biovolume of 48.5 µm, whereas drain sample 15C with the same *S. enterica* strain had the highest biovolume of 194.2 µm, in stark contrast to the control biofilms of 12C and 15C. Upon the introduction of diverse pathogen strains, variations in biofilm biovolume were noted. However, these changes did not elucidate a definitive trend of biofilm biovolume augmentation or reduction relative to the control. This suggests that while the presence of various pathogen strains could potentially affect the biovolume of mixed-species biofilms, the nature of these interactions remains unclear. Therefore, further investigation is imperative to delineate the specific influences of foodborne pathogens on the biovolume of mixed-species biofilms.

### Comparison of biovolume of mixed-species biofilms with and without enteric pathogen co-inoculation

The addition of *S. enterica* or *E. coli* O157:H7 strains to mixed-species biofilms resulted in variable changes in biovolume. Consistent increases in biofilm biovolume were observed in samples 9A, 11A, 8B and 15C, whereas consistent decreases were found in biovolumes for samples 8A, 9B, 12C and 17C (Figs. 14 and 15). *S. enterica* did not substantially change the biovolume of the mixed-species biofilm observed in sample 13C. Biovolume in sample 14C increased by 39.7%, 68.6%, and 18.2%, respectively, after the addition of *S. enterica* strains Cerro, Typhimurium, and Montevideo. Biovolume in sample 18C decreased by 24.5% and 17.3% when inoculated with *S. enterica* serovar C-Cerro and C-Montevideo, respectively, but increased by 35.9% when inoculated with *S. enterica* serovar C-Typhimurium. When *E. coli* O157:H7 strains 110 and 138 were inoculated into sample 10A, biovolume increased by 49.1% and 32.6%, respectively, while inoculation with strain 141 slightly decreased biovolume by 3.1%. Similarly, the addition of *E. coli* O157:H7 strain 110 increased the biovolume of sample 10B by 16.1%, whereas the addition of strains 138 and 141 decreased the biovolume by 32.6% and 40%, respectively. In contrast, the biovolume of sample 11B decreased by 44.3% when *E. coli* strain 110 was introduced but increased by 38.1% and 20.0% when *E. coli* 138 and 141 were introduced, respectively. The results suggest that the biovolume of mixed-species biofilm can be influenced by the strain of the pathogen, with some strains causing an increase in biovolume and others causing a decrease. Additionally, the results indicate that even within the same drain sample, different pathogenic strains can have varying effects on biovolume. However, based on the statistical analysis, it was determined that none of these changes were statistically significant (*p *> 0.05), possibly as a result of the study's limited sample size.

### Growth curve of S. enterica and E. coli O157:H7 pathogen in mixed-species biofilms

The growth of *E. coli* O157:H7 and *S. enterica* cells in each single-strain biofilm or in mixed biofilms formed with drain samples were measured at 7 °C for 24, 48, 72, 96, and 120 h. Overall, the densities of *E. coli* O157:H7 and *Salmonella* biofilm cells reached approximately 7.1–8.0 and 5.5–6.3 Log CFU/mL, respectively. Importantly, both pathogens exhibited minimal growth as statistical analysis showed no significant cell density change by comparing the five-time points tested. For each strain, no significant difference in the pathogen cell densities was observed among its single-strain biofilm and the mixed biofilms at each testing time point as well as over the course of the 5 days.

### Verification of BacLight Red Stain staining effectiveness for bacterial cells

The verification process involved staining *E. coli* and *S. enterica* cultures at 37° C with *BacLight* Red Stain overnight. The overnight stained bacterial culture where centrifuged and separated as bacterial pellets and suspend medium. The observations revealed that only the bacterial cells in the pellet were stained, confirming the effectiveness of the staining process. Therefore, it was concluded that the stain effectively tagged only the desired bacterial cells (Supplementary Fig. 1).

## Discussion

This study aimed to develop a novel method for tracking the presence and location of foodborne pathogens in mixed-species biofilms using the DSFM technique. In this study, three strains each of *S. enterica* and *E. coli* O157:H7 were examined within mixed-species biofilms formed from drain samples collected from beef processing facilities.

The populations of *S. enterica* and *E. coli* O157:H7 cells in mixed-species biofilms exhibited minimal growth, both as individual strains and when co-inoculated into the drain samples. This observation suggests that these bacteria were in a stationary phase. According to Harms et al. and , many bacterial species form biofilms under non-optimal growth conditions, and biofilm bacteria share physiological similarities with bacteria in the stationary phase^11,12^. Despite the cold temperature limiting the growth of *E. coli* and *S. enterica*, these bacteria could still contribute to biofilm formation. Therefore, the observed increase in biovolume in certain drain samples which linked to an increase in biofilm formation as a survival strategy under non-optimal conditions, rather than an increase in the bacterial population. The limited growth observed under these conditions, while likely due to the low temperature, also allowed for reliable tracking of the stained pathogen throughout our experiment. If significant pathogen growth had occurred, the loss of dye in newborn cells could have complicated this tracking process and potentially impacted our results. The optimal growth temperature for both *S. enterica* and *E. coli* O157:H7 is 37 °C. However, at 7 °C, the metabolic processes of these bacteria become less efficient, leading to diminished growth and cell division rates. The low temperature impacts various aspects of bacterial physiology, including the fluidity of cell membranes, the functionality of enzymes involved in essential cellular processes, and the efficacy of nutrient transport into the cell^13^. Consequently, *S. enterica* and *E. coli* O157:H7 encounter difficulties replicating under these conditions. Also, competition between species or suppression by other bacteria may have played a role. These complex drain biofilm samples contained high microbial diversity^14,15^. The interspecies interactions would have a significant role in determining the distribution of different species members in the mixed-species biofilm due to the fact that each species has its preferences in terms of temperature, pH, and other factors^16^.

The *BacLight* Red Bacterial Stain was used to track enteric foodborne pathogens and FM-1-43 to counterstain the mixed-species biofilms. Their distinct advantages determined the selection of these two stains in cell biology research. The *BacLight* bacterial stain was chosen for its efficacy and several key advantages. It offers a rapid staining protocol, capable of marking both live and fixed bacteria within a 15-min window, thus facilitating high-throughput analysis. It can be used with live or fixed cells and does not bind to the DNA which could potentially alter bacterial biological processes^17^.The stain emits a bright red fluorescence, providing high-contrast visualization and specificity by selectively staining bacterial cells, thereby minimizing background interference. Its widespread use in bacterial imaging and quantification applications further attests to its utility^17^. When compared to traditional chromogenic stains, which require multiple-step preparation and longer incubation, *BacLight* stands out with its rapid kinetics, fluorescence signaling, and specificity for bacterial cells. These attributes make it particularly well-suited for efficiently detecting and analyzing pathogens within complex multispecies biofilms. FM-1-43 is a fluorescent dye that can selectively stain cell membranes without affecting cellular processes. Previous studies have confirmed its ability to stain bacteria^3^. Its compatibility with other fluorescent markers makes it useful for studying membrane dynamics in mixed-species biofilms. *BacLight* Red Bacterial Stain provides high effectiveness for bacterial cells, allowing tracking of pathogens in the presence of other organisms. Both stains require minimal preparation, making them safe and reliable options for bacterial studies. The combination of *BacLight* Red Bacterial Stain and FM-1-43 is an effective and efficient approach to directly visualize interactions between foodborne pathogens and mixed-species biofilms^3^.

By using DSFM method, the results revealed that *E. coli* O157:H7 and *S. enterica*, are predominantly located in the middle and upper layers of the mixed-species biofilms (Figs. 2, 3, 4, 5, 6, and 7). The presence of zones of active growth in the different layers of biofilm is highly dependent on the specific metabolism of a given species, specifically whether oxygen is required for growth or not^18^. As facultative anaerobes, *E. coli* and *S. enterica* remain on the top layer, which not only allows them to exist in an oxygen-and nutrient-rich environment and perform aerobic respiration, but also permits them to expand to other areas one order of magnitude faster than the bacteria on the bottom layer^19,20^. However, remaining at the surface also comes with a drawback, as these bacteria are more susceptible to exposure to sanitizers and other antimicrobial agents. This observation has important implications for understanding pathogen persistence within biofilms and for developing targeted cleaning and disinfection strategies in beef processing facilities.

Previous research has shown that mixed-species biofilms are more resistant to cleaning and disinfection methods than single-species biofilms, due in part to their increased structural complexity and variety of protective mechanisms^21^. Various factors, including nutrient availability, oxygen gradients, and interspecies interactions, can influence the spatial distribution of foodborne pathogens within mixed-species biofilms^22–24^. Nakanishi et al. showed that the spatial localization dual-species biofilms were altered in a manner that depended on the partner organism, while Bridier et al. redemonstrated how interspecies interactions within mixed-species biofilms affected the spatial organization and resistance to antimicrobial treatments^18,20^. Flemming et al. discussed the complexity of biofilm matrix and its surroundings can impact pathogen behaviors^19^. The presence of pathogens in the biofilm's top or middle layers may indicate their preference for specific microenvironments, such as those with higher nutrient availability or optimal oxygen levels. This spatial distribution pattern of pathogens can be connected with the total biovolume of the mixed-species biofilm matrix and their sanitizer tolerance level^14^. *E. coli* O157:H7 cells in mixed biofilms obtained from a beef processing plant with recurrent *E. coli* O157:H7 prevalence displayed significantly greater sanitizer tolerance than those obtained from a control plant^3^. Consequently, the dispersal of biofilms in beef processing facilities may result in downstream food contamination, posing a potential risk to food safety^25,26^.

Our results highlight the differences in biofilm formation between drain samples. These findings confirm the ability of microorganisms to form biofilms on SS surfaces under conditions relevant to the food processing industry. Furthermore, when co-inoculating the drain samples with *E. coli* O157:H7 or *S. enterica*, the mixed-species biofilms demonstrated even greater variability in biovolume (Figs. 14 and 15). This suggests that the specific strains of foodborne pathogens may have different effects on biofilm formation on SS surfaces, and that different results can be observed for samples from the same beef processing facility. The observed strain-specific effects on biofilm structure and biovolume may be attributed to various factors, including differences in adhesion, biofilm matrix production, and inter-species interactions. For example, some strains of *E. coli* and *S. enterica* may produce more extracellular polymeric substances (EPS), enhancing biofilm formation and structure^27,28^. Additionally, certain strains may exhibit enhanced adhesion capabilities, allowing them to better integrate into the mixed-species biofilm^29,30^. The drain samples used in this study were previously characterized with 16S rRNA analysis. The potential correlation between the presence of the various species compositions and the spatial distribution of the pathogen cells in the mixed biofilms is beyond the scope of this study but warrants future investigation. Our findings have practical implications for the food industry, particularly in the design of targeted control strategies for mixed-species biofilms in beef processing facilities. By understanding the strain-specific effects on biofilm properties, more effective cleaning and disinfection protocols can be developed, addressing the unique challenges posed by different foodborne pathogen strains.

The findings of this study provide valuable insights into the location and biovolume of foodborne pathogens in mixed-species biofilms, paving the way for future research directions. An alternative course of action that could be taken is to enhance the methodology employed in the study, which was specifically designed to track enteric pathogens in cold processing environments. While this tactic did provide significant observations on the proliferation and growth of pathogens within mixed-species biofilms at different temperatures, it would be beneficial to widen the scope of this research to encompass other ecological constituents. This would allow for a more comprehensive assessment of the applicability of our findings. Another area for future exploration is the impact of sample size on the biovolume results. In this study, the small sample size may have limited our ability to detect significant changes. By increasing the sample size in future studies, we could potentially track more subtle differences and obtain more robust statistical results. This would provide more conclusive evidence of the impact of staining dyes on pathogen growth and biovolume. Finally, it would be worthwhile to explore the use of alternative or complementary staining techniques. This could enable simultaneous tracking of different pathogens within the same biofilm, providing a more comprehensive understanding of the interactions between various foodborne pathogens. Such knowledge would enhance our ability to develop targeted strategies for controlling pathogen proliferation in mixed-species biofilms. In particular, the use of the *BacLight* Red Stain, which may not transmit to newly divided cells, could be reevaluated or supplemented with other stains to improve the accuracy of our tracking methods.

In conclusion, this study establishes differential staining fluorescence microscopy (DSFM) as an effective approach for investigating the spatial distribution of foodborne pathogens within mixed-species biofilms. The findings suggest that the presence of mixed-species biofilms in a beef processing plant environment may contribute to the contamination of beef products with pathogens such as *E. coli* O157:H7 and *S. enterica*. Using DSFM shows the importance of considering the spatial distribution of pathogens within a mixed-species biofilm, as this can have implications for the effectiveness of sanitizers and other control measures. In essence, our innovative method not only provides a way to locate and track pathogen distribution within complex biofilms but also offers valuable insights into the dynamics of biofilm formation and development under cold-chain food processing facilities. These findings demonstrate the utility of DSFM for studying biofilms relevant to food safety and highlight the importance of considering pathogen distribution when developing targeted control strategies. Further application of this novel technique will continue to advance the understanding of mixed-species biofilm complexity and the role of pathogens within these communities.

## Methods

### Drain sample and foodborne pathogens collection

Fourteen floor drain samples were obtained from coolers and hotboxes at three beef processing plants A, B and C. Hotbox drains were those in the portion of the beef processing plant where carcasses that immediately exit the harvest floor are spray-chilled to ~ 1 °C and stored for 24–48 h. Cooler drains were those in the portion of the beef processing plant where carcasses are graded, sorted, and stored at ~ 1 °C before fabrication. Sample collection was conducted as previously described. Briefly, Buffered peptone water wetted Speci-Sponges (Whirl–Pak, Madison, WI) were used to vigorously swab the interior of opened floor drains to collect any surface-bound bacteria and biofilm (slimy coating or layer) visible. Sponge samples were transported to the lab in insulated coolers with wet ice packs. Samples were homogenized by hand massaging, and portions were aliquoted to freezer vials. The aliquoted samples were stored at − 80 °C with 17% glycerol as a cryopreservative. Three *E. coli* O157:H7 strains were previously isolated from “High Event Period” trim contamination at commercial beef processing plants^31^. Three *S. enterica* strains were previously isolated from Plant C floor drains^15^. Strains Cerro and Montevideo were isolated from hotbox drains, and strain Typhimurium was isolated from a processing floor drain. The three *E. coli* O157:H7 isolates were obtained from Plants A and B following historical *E. coli* contamination events at those facilities. This selective isolation enabled the investigation of the most epidemiologically relevant pathogens for each plant. The *E. coli* O157:H7 and *S. enterica* strains were isolated from enriched trim or floor drain samples by immunomagnetic separation then streaked onto selective agar plates, CHROMagar-O157 and XLD for O157 and *Salmonella*, respectively. Suspect colonies were confirmed by multiplex polymerase chain reactions and serotyped using slide agglutination and tube agglutination methods as previously described.

### Culture conditions for drain samples, and S. enterica and E. coli O157:H7 strains

To simulate the drain environment and to preserve the microbial composition of the fourteen floor drain samples, each frozen sample from above was 50-fold inoculated into the Luria Bertani No Salt (LB-NS) medium and incubated at 7 °C for 5 days with orbital shaking at 200 rpm. The expanded samples were aliquoted and stored at − 20 °C in LB-NS medium containing 15% sterile glycerol. The six strains of food-borne pathogens: *S. enterica* serovar strains C-Montevideo, C-Typhimurium and C-Cerro were isolated from floor drains at processing plant C, and *E. coli* O157:H7 strains: 110, 138 and 141 were obtained from naturally contaminated ground beef. The six strains were kept at − 80 °C in LB-NS medium supplemented with 17% glycerol. For each experiment, the strains were revived from − 80 °C by inoculating into Tryptic Soy Broth (TSB) (Difco, Beckton Dickinson, Sparks, MD) and incubating at 37 °C for 18 hours^1^.

### Drain sample biofilms and mixed-species biofilm formation for fluorescence microscopy scanning

To develop mixed-species biofilms for fluorescence microscopy scanning, the drain samples were inoculated in LB-NS at 7 °C for 5 days with orbital shaking at 200 rpm. The three *S. enterica* and three *E. coli* O157:H7 strains were individually inoculated with *BacLight* Red Bacterial Stain (ThermoFisher Scientific, Germany) (1:1000 in filter-sterilized water). The stained cultures were grown for 18 h in LB-NS at 37 °C prior to the start of construction on site. Six-well plates (VWR, USA) containing sterile SS chips were employed as the base material for developing the mixed-species biofilms. The 5-day drain culture and overnight *S. enterica* and *E. coli* pure culture were incubated together on SS chips submerged with LB-NS media for 5 days at 7 °C (Fig. 1). Additionally, drain biofilms without *S. enterica* and *E. coli* inoculations were also developed with the same condition for biovolume comparison.

### Fluorescent microscopy 3D scanning

After 5 days of incubation, SS chips with mixed-species biofilms containing the strains of *S. enterica* or *E. coli* developed on the surfaces were counterstained with FM 1-43 dye (1:1000 in filter-sterilized water) for 15 min at room temperature in the dark (Life Technologies, Eugene, Oregon, USA) before imaging. The excess dyes were removed, and the samples were kept in filter sterilized water. Imaging was done using fluoresce microscopy GFP filter which has an excitation wavelength of 450 to 490 nm and an emission wavelength of 500 to 550 nm. The magnification was achieved using the object lens HC PL FLUOTAR 20x/0.55 PH2. Analysis of the 3D structural organization of the biofilms and the superimposed images of both filters were carried out by the Leica Application Suite X (version 3.7.2). The biovolumes were obtained using the Leica 3D Analysis Module package by averaging the values obtained from nine distinct points in the center of the biofilms on the SS chip. This experiment was repeated twice.

### S. enterica and E. coli O157:H7 growth curves at 7 °C in presence of expanded drain samples

Overnight culture of each *E. coli* or *S. enterica* strain were grown by inoculating 50 µL of frozen glycerol stock into 15 mL of TSB, followed by overnight incubation at 37°C under static conditions. The next day, biofilm was set up by mixing 50 μL of overnight bacterial culture, 100 μL of biofilm enrichment culture, and 3.85 mL of LB-NS. The biofilm enrichment culture used were frozen stocks in glycerol prepared as described above in culture conditions for drain samples methods. For pathogen-only cultures, biofilm enrichments were substituted with 100 μL of LB-NS. The cultures were incubated at 7 °C under static conditions for 5 days. During incubation, 100 μL of cultures were aliquoted at every 24 h, then serially diluted in normal saline, and plated onto Chromagar O157 (DRG Industries, Springfield, NJ) plates or Remel Xylose, Lysine, Desoxycholate (XLD) agar (Thermo Scientific, Waltham, MA) plates for enumeration of viable cells of *E. coli* or *S. enterica*, respectively*.* A dilution of 10^–4^ was found to be optimal for enumerating viable cells. The plates were incubated at 37 °C for 24 h.

For statistical analysis, bacterial colony forming units (CFUs) were transformed to log CFU/mL and average log CFU/mL was calculated from three replicates. Computation of permutation p-values and pairwise comparison between different incubation time points for each strain growing alone as well as in mixed culture with biofilm samples was performed using compareGrowthCurves (CGGC permutation test) function of the statmod R package^32^.

### Verification of BacLight Red Stain staining effectiveness

One milliliter of each S*. enterica* or *E. coli* O157:H7 culture was separately stained with *BacLight* Red Stain and incubated overnight. After incubation, the stained cultures were centrifuged at 10,000 × g for 5 min to divide them into pellet and supernatant fractions. The supernatants were further purified using a 0.45 µm filter to guarantee staining effectiveness. Both the pellet and supernatant fractions were then introduced separately into the biofilm on the SS chip. Fluorescence microscopy was employed to capture images of the sample, utilizing a Y5 filter with an excitation wavelength of 590–650 nm and an emission wavelength of 662–738 nm. A HC PL FLUOTAR 20x/0.55 PH2 objective lens was used to achieve magnification. The Leica Application Suite X (version 3.7.2) was utilized for the analysis of the 3D structural organization of the biofilms and superimposed images of both filters. The Leica 3D analysis module package was employed to obtain the biovolume. This procedure aimed to confirm the effectiveness of *BacLight* Red Stain for bacterial cells and eliminate any background signal that could affect the imaging results.

### Supplementary Information

Supplementary Figures.

## Data Availability

All data generated or analysed during this study are included in this published article and supplementary data (1).
