# Supplementary material for: A novel method using a differential staining fluorescence microscopy (DSFM) to track the location of enteric pathogens within mixed-species biofilms
Source: Sci Rep. 2023 Sep 16;13:15388. doi: 10.1038/s41598-023-42564-6 (PMC10505192; doi:10.1038/s41598-023-42564-6)
Supplement: Supplementary file 1 — Supplementary Figures. [file 41598_2023_42564_MOESM1_ESM.docx]

SUPPLEMENTARY


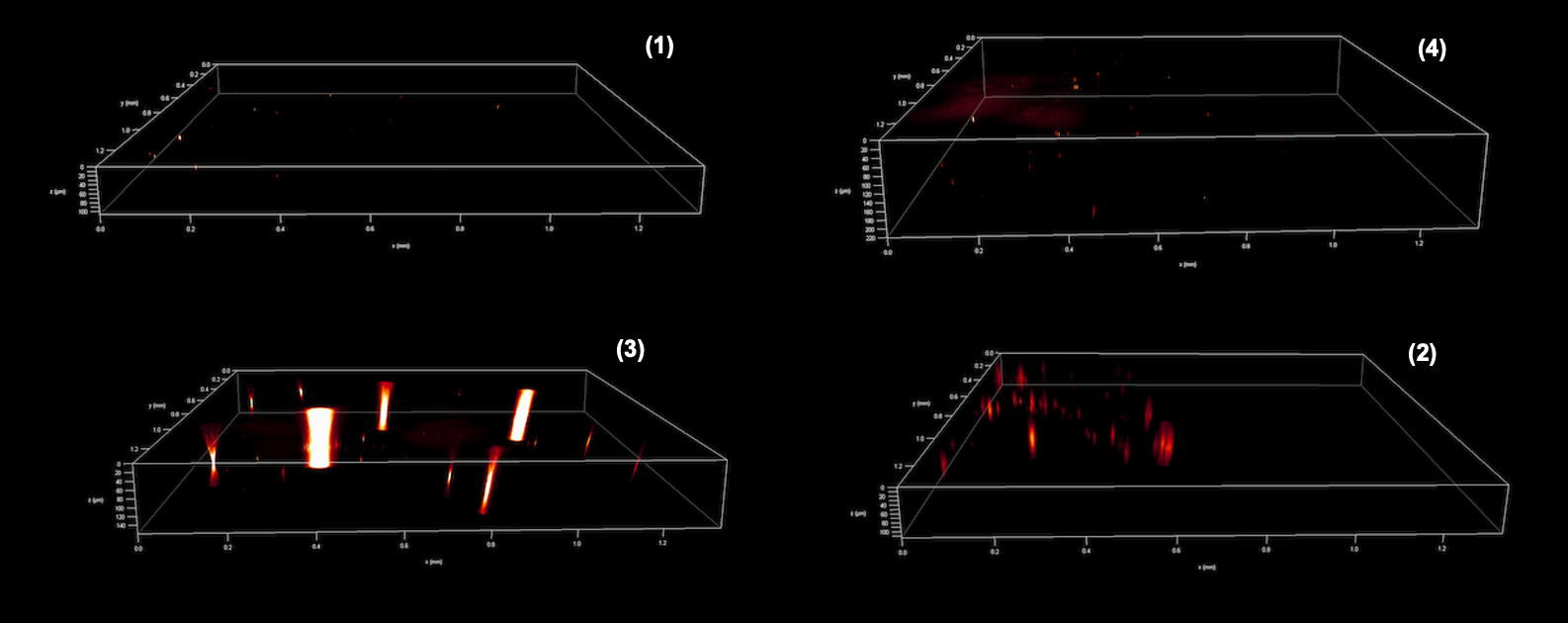


Fig. 1. Fluorescence images (magnification 10x) after all the samples were stained with *Bac*Light™ Red Bacterial Stain. (1) Control sample only contain LB-NS broth exposed under Y5 channel. (2) Supernatant of E. coli serovar 110 and sample 8A biofilm exposed under Y5 channel. (3) Pellet of E. coli serovar 110 and sample 8A biofilm exposed under Y5 channel. (4) E. coli serovar 110 and sample 8A biofilm exposed under Y5 channel.


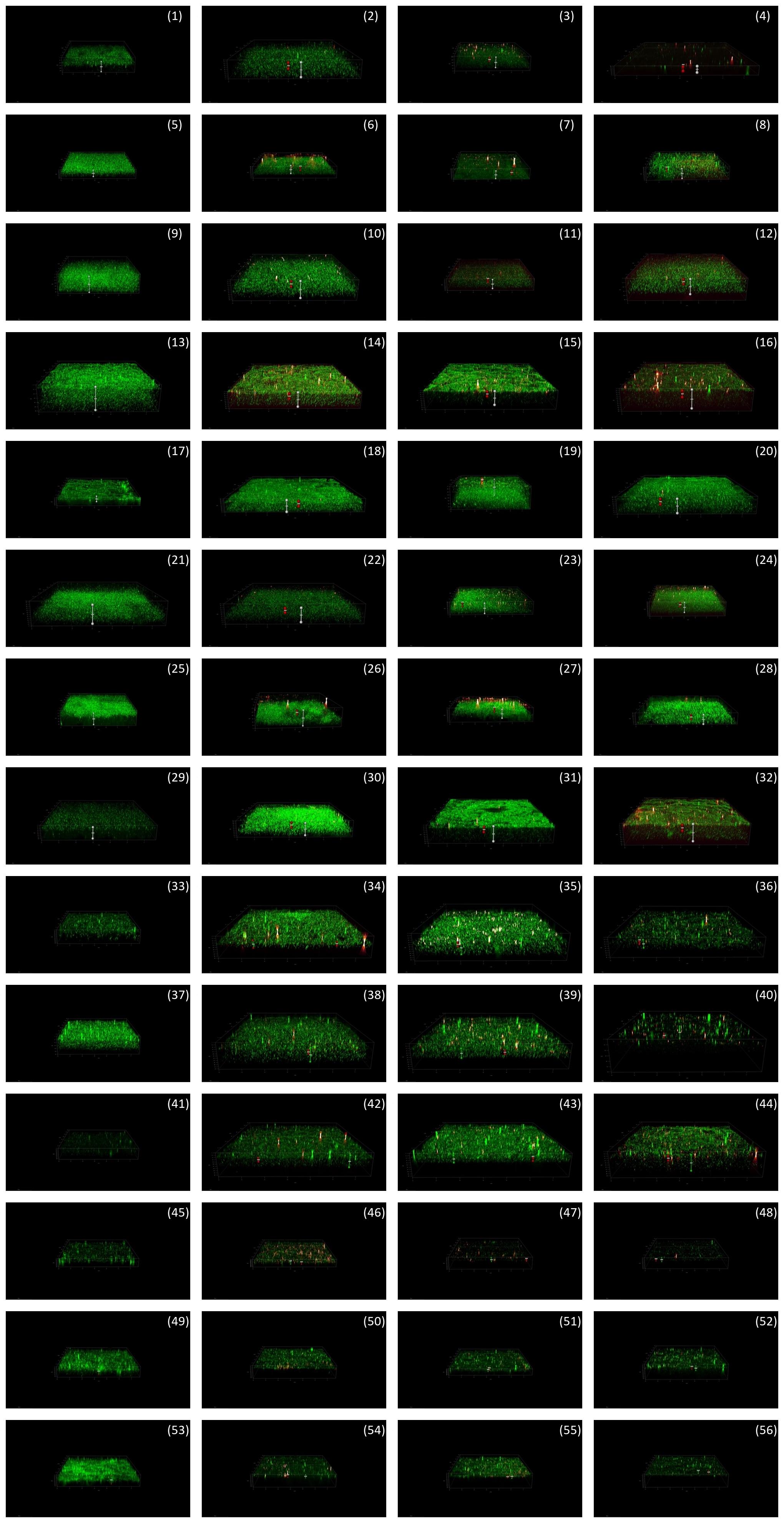


Fig. 2. Fluorescence images (magnification 10x) of

(1-4) 8A drain biofilm, and 8A drain biofilm with three different *E. coli* serovar (1)110, (2)138 and (3)141 exposed under both GFP and Y5 channel.

(5-8) 9A drain biofilm, and 9A drain biofilm with three different *E. coli* serovar (1)110, (2)138 and (3)141 exposed under both GFP and Y5 channel.

(9-12) 10A drain biofilm, and 10A drain biofilm with three different *E. coli* serovar (1)110, (2)138 and (3)141 exposed under both GFP and Y5 channel.

(13-16) 11A drain biofilm, and 11A drain biofilm with three different *E. coli* serovar (1)110, (2)138 and (3)141 exposed under both GFP and Y5 channel.

(17-20) 8B drain biofilm, and 8B drain biofilm with three different *E. coli* serovar (1)110, (2)138 and (3)141 exposed under both GFP and Y5 channel.

(21-24) 9B drain biofilm, and 9B drain biofilm with three different *E. coli* serovar (1)110, (2)138 and (3)141 exposed under both GFP and Y5 channel.

(25-28) 10B drain biofilm, and 10B drain biofilm with three different *E. coli* serovar (1)110, (2)138 and (3)141 exposed under both GFP and Y5 channel.

(29-32) 11B drain biofilm, 11B drain biofilm with three different *E. coli* serovar (1)110, (2)138 and (3)141 exposed under both GFP and Y5 channel.

(33-36) 12C drain biofilm, and 12C drain biofilm with three different *S. enterica* serovar (1) Cerro, (2) Montevideo and (3) Typhimurium exposed under both GFP and Y5 channel.

(37-40) 13C drain biofilm, and 13C drain biofilm with three different *S. enterica* serovar (1) Cerro, (2) Montevideo and (3) Typhimurium exposed under both GFP and Y5 channel.

(41-44) 14C drain biofilm, and 14C drain biofilm with three different *S. enterica* serovar (1) Cerro, (2) Montevideo and (3) Typhimurium exposed under both GFP and Y5 channel.

(45-48) 15C drain biofilm, and 15C drain biofilm with three different *S. enterica* serovar (1) Cerro, (2) Montevideo and (3) Typhimurium exposed under both GFP and Y5 channel.

(49-52) 17C drain biofilm, and 17C drain biofilm with three different *S. enterica* serovar (1) Cerro, (2) Montevideo and (3) Typhimurium exposed under both GFP and Y5 channel.

(53-56) 18C drain biofilm, and 18C drain biofilm with three different *S. enterica* serovar (1) Cerro, (2) Montevideo and (3) Typhimurium exposed under both GFP and Y5 channel.
